# Facilitating Cross-border Viral Sequencing Through Nucleic Acid Sample Transport Using Dry Cards

**DOI:** 10.3390/v17060804

**Published:** 2025-05-31

**Authors:** Lili Wang, Qikai Yin, Alie Brima Tia, Fengyu Tian, Liping Gao, Kai Nie, Kang Xiao, Xuejun Ma, Xiaoping Dong, Doris Harding, Xiaozhou He, George F. Gao

**Affiliations:** 1 National Institute for Viral Disease Control and Prevention, Chinese Center for Disease Control and Prevention, Beijing 102206, China; wangll@chinacdc.cn (L.W.); yinqk@ivdc.chinacdc.cn (Q.Y.);; 2 Center for Global Public Health, Chinese Center for Disease Control and Prevention, Beijing 102206, China; 3 Sierra Leone-China Friendship Biological Safety Laboratory, Ministry of Health and Sanitation, Freetown P.O. Box 232, Sierra Leone; tiaalie1@gmail.com; 4 Ministry of Health and Sanitation, Freetown P.O. Box 232, Sierra Leone; 5 CAS Key Laboratory of Pathogen Microbiology and Immunology, Institute of Microbiology, Chinese Academy of Sciences (CAS), Beijing 100045, China

**Keywords:** viral sequencing, dry cards, sample transport, stability

## Abstract

(1) Background: A safe and effective nucleic acid sample transportation method was developed that is suitable for underdeveloped areas which lack advanced sequencing capabilities, specifically for virus genomic sequencing and infectious disease monitoring. (2) Methods: This study evaluated the use of Flinders Technology Associates (FTA) cards for transporting amplified whole-genome DNA from 120 SARS-CoV-2-positive nasopharyngeal swab samples in Sierra Leone. Nucleic acid extraction and whole-genome amplification were conducted at a local laboratory. Amplified products were applied to FTA Elute cards for room temperature shipment to China CDC for elution and sequencing. (3) Results: The FTA card method achieved a 9.6% recovery rate for amplicons, sufficient for viral genome sequencing. In total, 86 (71.7%) high-quality SRAS-CoV-2 genomic sequences were obtained, with the majority reaching depths exceeding 100X. Sequence analysis revealed co-circulation of Delta, Omicron, and B.1 lineages. Higher Ct values in the original sample significantly reduced coverage and depth, with Ct ≤ 27; 73.6% of samples yielded effective sequences. (4) Conclusions: Transportation of amplified nucleic acid samples using FTA cards enables virus genomic sequencing in resource-limited areas. This approach can potentially improve local virus surveillance and outbreak response capabilities. Further optimizations could improve sequence recovery rate. Implementing this method could significantly enhance sequencing accessibility in underdeveloped regions.

## 1. Introduction

The COVID-19 pandemic has challenged global disease prevention efforts. Though viral genome sequencing is critical for outbreak response, resource-poor and remote settings often hinder its accessibility [1,2]. These limitations particularly impact comprehensive variant monitoring, which is crucial for mitigating evolving threats like SARS-CoV-2 [3]. Alternate transportation methods are needed to enable sequencing while ensuring biosafety [4].

Filter paper is often used to transport biological samples given its simplicity and stability, such as dried blood or other fluid sample spots [5]. The dried spots show compatibility with a wider range of pathogen detection [6,7]. However, the amount of nucleic acids extracted from samples on the card is generally low, making it primarily suitable for PCR or similar detection and challenging to use for genome sequencing [8,9]. Furthermore, dried spots also have limited biosafety capabilities against highly infectious samples [10]. Nevertheless, the utility of Flinders Technology Associates (FTA) cards for viral sequencing from remote areas has not been evaluated [11,12].

In collaboration between Sierra Leone and China, the Sierra Leone–China Friendship Biosafety Laboratory conducted 131,862 SARS-CoV-2 nucleic acid tests between 2020 and April 2023, accounting for 34.37% of all local tests in Sierra Leone. However, local virus genome sequencing is still limited due to a lack of reliable professionals, stable power supply, and suitable operating environments for sequencing equipment in local laboratories. This creates a significant gap in understanding the local COVID-19 epidemic situation [13]. To gain a comprehensive understanding, we established a complete workflow enabling viral sequencing from remote regions. This approach could significantly contribute to global surveillance efforts and enhance local outbreak response preparedness, particularly in underdeveloped regions.

## 2. Methods

### 2.1. Sample Collection and Processing in Local Laboratory

**Figure 1:**
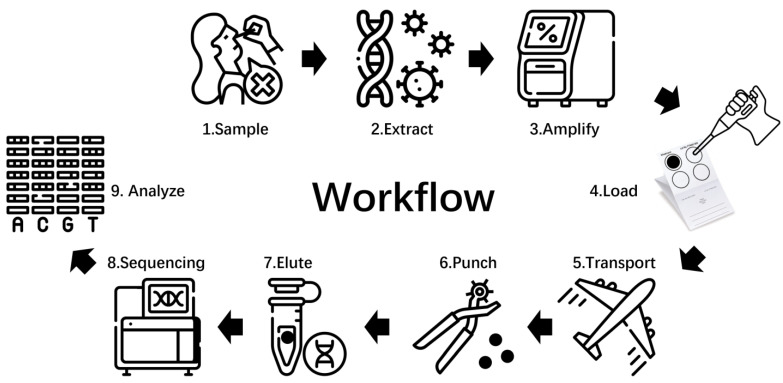
The overall workflow diagram comprises three main components: on-site work, FTA card sample transportation, and domestic laboratory sequencing.

The method includes three parts: fieldwork, sample transfer, and in-country laboratory procedures (Figure 1). A total of 120 SARS-CoV-2-positive nasopharyngeal swab samples were collected from individuals diagnosed between April 2021 and October 2022. The samples were stored at −40 °C prior to analysis. Nucleic acid extraction was performed using the Nucleic Acid Extraction System SSNP-3000A (Bioperfectus, Taizhou, China) and the Viral Nucleic Acid Extraction Kit (Magnetic Bead Method) (Cat. No: SDK60104D-32T, Bioperfectus, China), followed by whole-genome amplification using the Vazyme RNA Multi-PCR Library Prep Kit (Cat. No: NA211). Unpurified amplified products were directly quantified using the Qubit nucleic acid quantification kit (Thermo Fisher Scientific, Waltham, MA, USA), without additional cleanup steps to simulate field conditions. The sampling and experimental procedures of this study were reviewed by the institutional review board of Chinese Center for Disease Control and Prevention (China CDC) (Approval Notice No.202113) and Office of the Sierra Leone Ethics and Scientific Review Committee.

### 2.2. Loading DNA Product to FTA Card and Transportation

Amplified nucleic acids (50 μL per sample) were manually spotted onto individual FTA Elute cards (WB120410, Qiagen, Hilden, Germany). Cards were dried overnight at room temperature (25 °C) under ventilated conditions, then air transported to the laboratory of China CDC at room temperature under dry conditions for further processing. Four blank FTA card controls were included to monitor environmental contamination.

### 2.3. Nucleic Acid Elution and Sequencing

Four 6 mm punches covering the sample area were taken from each FTA Elute card with a clean punch and placed into a single 2 mL microcentrifuge tube. Nucleic acids were eluted from the punches using TE buffer (Sangon, Shanghai, China) and QIAcard FTA Elute Buffer (Qiagen, Germany) according to its manual. Ultimately, the nucleic acids were dissolved in 250μL Elute Buffer and saved for subsequent sequencing. The eluent was re-quantified using Qubit (Thermo Fisher Scientific, Waltham, USA) and confirmed via RT-qPCR (Bioperfectus, Taizhou, China). Nucleic acid samples passing quality control were constructed into libraries and sequenced on the NovaSeq platform (Illumina, San Diego, CA, USA) with 150 bp length and paired-end sequencing. Nucleic acid samples proceeding to library preparation met dual quality thresholds: (1) Qubit-quantified concentration > 1 ng/μL in ≥15 μL elution volume and (2) Bioanalyzer (2100, Agilent, Santa Clara, CA, USA)-verified fragment distribution showing the expected ~400 bp profile.

### 2.4. Data Analysis

Raw reads were quality-filtered using fastp (v0.23.4) then aligned to SARS-CoV-2 reference genome (NC_045512.2) with bowtie2 (v2.3.5.1). Alignment processing, variant calling, calculation of the sequencing depth and coverage, and consensus generation were conducted in Samtools (v1.17). Sequence lineages were assigned via phylogenetic placement using Nextclade (v2.14.1 https://clades.nextstrain.org/ (accessed on 17 August 2023)) and Pangolin (v4.3, https://pangolin.cog-uk.io/ (accessed on 17 August 2023)). Statistical analysis was performed using GraphPad (v.9.0.0, GraphPad Software, USA) with a significance level of 0.05.

## 3. Results

### 3.1. Nucleic Acid Recovery from FTA Card

**Figure 2:**
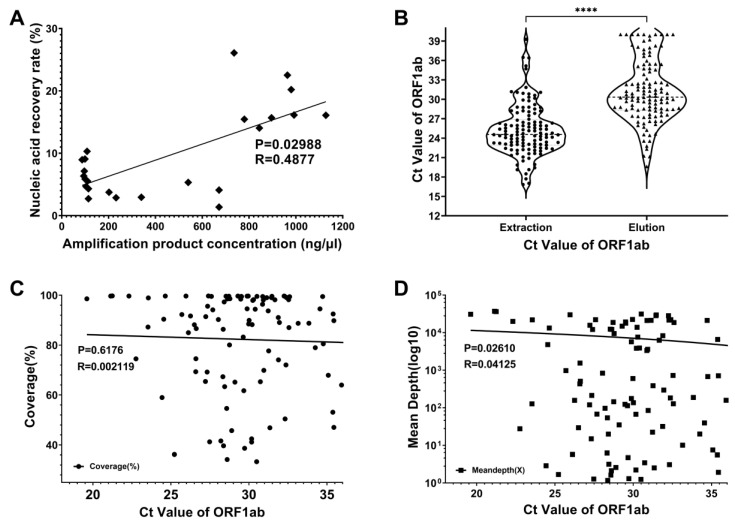
The main factors that influence the success rate and sequencing quality. (**A**). The correlation between nucleic acid concentration and sample recovery efficiency. (**B**). Comparing on-site extracted nucleic acids and FTA-card-eluted nucleic acids with SRAS-CoV-2 rt-qPCR results. (**C**). The correlation between genomic coverage and Ct value. (**D**). The correlation between genomic sequencing depth and Ct value. “****” indicate a highly significant *p*-value (*p* <= 0.0001).

The average concentration of initial amplification products was 458.5 ng/μL (95% CI: 299.2 to 617.9). After FTA elution, concentration dropped to 12.1 ng/μL (95% CI: 5.4 to 18.8). The average recovery rate of nucleic acid products was determined to be 9.6% (95% CI: 6.7% to 12.6%). A positive correlation was observed between the recovery rate and initial amplification product concentration (Figure 2A). Cycle threshold (Ct) values of eluted nucleic acid (mean 31.16, 95% CI: 30.30–32.03) were significantly higher (*p* < 0.001, Figure 2B) than direct detection in Sierra Leone (mean 25.2, 95% CI: 24.51–25.93). Thus, FTA eluates showed significantly higher Ct values compared to initial extracts (*p* < 0.001, Figure 2B), indicating nucleic acid loss. It can be inferred that higher initial amplicon concentration leads to better recovery after FTA elution. Although 34 samples were deemed as sequencing-failed due to <70% coverage relative to the reference genome, data showed their coverage ranged from 33.3% to 69.2%, with sequencing depths of 1.2X to 432.2X. Real-time PCR results revealed Ct values of 24.4–40.0, where 31 samples (91.2%) had Ct values > 27, indicating possible viral RNA degradation in original samples. The remaining three cases might be due to technical errors leading to low nucleic acid recovery.

### 3.2. Sequencing Performance

A total of 1125.3 million reads were generated from all samples, averaging 9.4 million reads per sample (95 % CI: 8.5 to 10.3) and 1.4G data (95 % CI: 1.3 to 1.5). An average of 750,598 reads (95 % CI: 545,648 to 955,549) were aligned to SARS-CoV-2 genome. A total of 86 valid genomic sequences (71.7 %, coverage > 70%) and 59 complete genomes (49.2 %, coverage > 90%) were obtained. In total, 83.7 % (72 out of 86) of the effective genome sequencing had a depth of 100X or above. Coverage and depth decreased with increasing Ct values (Figure 2C,2D), with depth showing a significant negative correlation (*p* < 0.05). The results showed that Ct values have a greater impact on genome coverage and sequencing depth.

### 3.3. SARS-CoV-2 Lineage Classification

**Figure 3:**
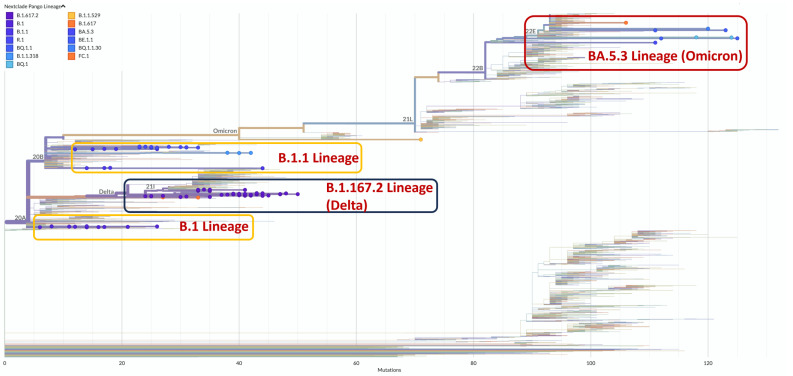
Phylogenetic analysis of SARS-CoV2 genome based on samples collected in Sierra Leone. A rectangular phylogeny tree (without roots) was constructed using Nextclade tools. Sequences from the present study were placed (colored dots) on a reference tree. The Nextstrain and WHO clade names are shown on the respective colored dots.

Phylogenetic analysis revealed 14 lineages across 86 high-quality genomes, dominated by Delta (*n* = 43), Omicron (*n* = 14), and B.1/B.1.1 variants (*n* = 29) (Figure 3). Despite global Omicron dominance since 2022, all three lineages persisted for a long period in Sierra Leone throughout sampling (Table 1). In this study, the sampling times of the local lineages detected were all after their discovery on a global level. Therefore, it is difficult to determine whether these lineages evolved locally or were imported externally. Furthermore, mutation sites were consistent with respective known strains, with no unique significant mutations identified.

## 4. Discussion

Pathogen genome sequencing is crucial for outbreak response and future epidemic preparedness. However, remote or underdeveloped areas face considerable challenges in performing this technique due to limited resources, technical expertise, and infrastructure [2,3]. While recent advancements like Nanopore sequencing enable on-site testing, their lower throughput and higher costs make them more suitable for initial outbreak phases than long-term monitoring or research. Transporting samples to well-equipped laboratories offers an alternative, but cross-regional or international transport raises biosafety concerns and ethical considerations [12]. Thus, it becomes imperative to develop a secure and efficient method to transport nucleic acid samples, supporting sequencing work on samples from these regions [4,5]. While enhancing the capacity for monitoring local infectious diseases, support will also be provided to improve global public health response capability [14].

This study demonstrates a complete workflow enabling viral genomic sequencing from remote, under-resourced regions. It achieves this by transporting amplified nucleic acids on FTA cards, which not only eliminate the need for strict cold chain transportation but also avoid the associated biosafety risks. Importantly, this method transports inactivated nucleic acids, ensuring that no live virus is involved, thus enhancing the safety of the transportation process. In addition, samples transported using the FTA card can be stored at room temperature for an extended period and, even when eluted at least 60 days later, they still support virus genomic sequencing. Some studies have also found that FTA cards have demonstrated effectiveness in preserving the stability of nucleic acids for long-term storage [15,16]. When the cards were stored at −80 °C or −20 °C, vRNA was stable over 3 months compared to storage at 4 °C and room temperature [17].

Despite some sample loss, 86 effective SARS-CoV-2 genomes were recovered from Sierra Leone. Notably, co-circulation of Delta, Omicron, and B.1 lineages was observed even after 2022, which is significantly different from previous reports on the epidemic situation before November, 2021 [13]. The ongoing co-circulation of Delta and B.1 lineages may stem from low local vaccination rates and restricted cross-border population mobility, which delays variant replacement. Further analysis should be conducted in combination with epidemiological data.

There are differences in the quality of the recalled genomes. Both the sample Ct values and the recovery of nucleic acids from the FTA card are affected [8]. The results showed that, although the transportation of nucleic acids using FTA cards may result in fragment loss in some samples, the original Ct values of the samples have a more significant impact on genome coverage and sequencing depth, in line with expectation [4,8]. Therefore, we recommend FTA card sequencing for samples with Ct ≤ 27. In this range, 73.6% achieved effective sequencing (>70% coverage at >100X depth) and 52.7% yielded nearly complete genomes (>90% coverage). For samples with high initial Ct values (> 27), where standard FTA card processing yields limited sequencing success, standard frozen storage (−80 °C) of raw sample material may be preferred when logistics permit.

This study has several limitations. The sequencing success rate and quality of nucleic acid samples transported by FTA cards are lower compared to directly sequenced nucleic acids. Therefore, this method is only applicable when local sequencing is not feasible or when transporting original samples is inconvenient. Additionally, the sample size in this study was relatively small, limiting the ability to fully explain the local SARS-CoV-2 epidemic or determine whether variants were locally evolved or imported. While this study confirmed sequencing feasibility after 60 days of storage, further research should evaluate the impact of longer storage periods (e.g., 1 year) on nucleic acid integrity. The field-adapted protocol involving direct quantification of unpurified amplicons may have impacted the accuracy of nucleic acid recovery estimates. While this approach was adopted to maintain field applicability, future studies should include parallel purified controls to better quantify the actual nucleic acid recovery rates from FTA cards. Improvements to enhance recovery rates could include optimizing the elution buffer (e.g., adding carrier RNA), adjusting the number of punches (e.g., six holes per sample), or using vacuum-assisted elution techniques. Moreover, refining spotting techniques—such as optimizing volume, method (e.g., more uniform spotting approach), and drying conditions—could reduce nucleic acid loss and diffusion on FTA cards, thereby improving elution efficiency.

In conclusion, this study demonstrates the potential of FTA cards for reliable sample transport across regions. Future optimizations, such as improved nucleic acid recovery protocols, could enhance sequencing success and quality. This method also supports other applications like metagenomic sequencing, ultimately strengthening pathogen monitoring capabilities in underdeveloped areas [14]. By transporting inactivated nucleic acids, this approach not only avoids the safety concerns of live virus transportation but also mitigates the ethical issues associated with the direct transport of original samples or the extraction of nucleic acids from such samples. This will facilitate early threat detection and co-ordinated public health responses. This method can be extended to other viruses or bacterial genome monitoring, but amplification primers and elution procedures need optimization. For high-GC-content pathogens, modified buffers could be tried to improve recovery rates. While this study used FTA cards for field applicability, future work can adopt a paired-sample design including frozen controls. This will directly compare sequencing metrics between FTA and frozen samples to quantify FTA’s specific impact on nucleic acid recovery and sequencing performance.

## Figures and Tables

**Table 1:** The SARS-CoV-2 lineage obtained from sequencing and its collection time.

Lineage	Number of Seqes	Ancestral Lineage	WHO Clade	Collection Date
B.1.617.2	43		Delta	2021/12/25~2022/10/1
B.1.1.529	3		Omicron	2022/1/1
BE.1.1	2	BA.5.3	Omicron	2022/1/1, 2022/10/1
BQ.1	2	BA.5.3	Omicron	2022/7/5, 2022/7/26
BQ.1.1	4	BA.5.3	Omicron	2021/12/24~2022/1/1
BQ.1.1.30	1	BA.5.3	Omicron	2022/1/1
BQ.1.5	1	BA.5.3	Omicron	2021/12/25
FC.1	1	BA.5.3	Omicron	2022/10/1
B.1	2			2021/12/25, 2021/12/30
B.1.1	10			2021/12/26~2022/4/1
B.1.1.318	3	B.1.1		2022/2/1
R.1	14	B.1.1		2021/12/25~2022/1/1

## Data Availability

The data is deposited in National Microbiology Data Center (NMDC) with Accession Numbers NMDC10018794 (https://nmdc.cn/resource/genomics/project/detail/NMDC10018794). All virus sequences reported in this study were deposited in the NMDC under accession numbers NMDC60147339~NMDC60147458.

## Abbreviations

FTA, Flinders Technology Associates

Ct, Cycle Threshold
